# Haemostasis, inflammation and renal function following exercise in patients with intermittent claudication on statin and aspirin therapy

**DOI:** 10.1186/1477-9560-4-9

**Published:** 2006-07-18

**Authors:** Patrick Collins, Isobel Ford, Bernard Croal, Derek Ball, Michael Greaves, Ewan Macaulay, Julie Brittenden

**Affiliations:** 1 Department of Vascular Surgery, University of Aberdeen, UK; 2 Human physiology, University of Aberdeen, UK; 3 Department of Medicine and Therapeutics, University of Aberdeen, UK; 4 Vascular Unit, Aberdeen Royal Infirmary, Aberdeen, UK; 5 Department of biochemistry, Aberdeen Royal Infirmary, Aberdeen, UK

## Abstract

**Background:**

Previous studies have suggested that exercise in patients with intermittent claudication (IC) may induce a systemic thrombo-inflammatory response. The effect of secondary prevention therapy on this response is unknown. This study aimed to investigate the effects of treadmill exercise on markers of coagulation activation, inflammation and renal function in patients with IC, receiving aspirin and statin therapy compared to healthy controls.

**Methods:**

Samples were taken before, immediately and 1 hour after exercising on a treadmill in 20 patients with IC and 20 healthy volunteers. Interleukin-6 (IL-6), thrombin-anti-thrombin complex (TAT) and fibrin D-dimer were measured by ELISA. High sensitivity CRP (HsCRP) and urinary albumin were measured via a nephelometric technique, urinary protein via a turbidometric assay and N-acetyl-β-D-glucosaminidase (NAG) via a colorimetric assay.

**Results:**

Elevated baseline levels of Hs-CRP, IL-6, white cell counts, D-dimer and urinary NAG occurred in patients with IC compared to volunteers (p > 0.05). Following exercise there was no increase in Hs CRP or IL-6. D-dimer levels significantly increased following exercise in the patients and volunteers. TAT levels increased immediately after exercise in the patient group only and were significantly increased at 1 hour in both patients and volunteers. A transient rise in the protein creatinine ratio occurred in both groups (p < 0.007), and in albumin creatinine ratio in the patient group. There was no change in urinary NAG.

**Conclusion:**

Elevated markers of inflammation occurred in patients with IC on statin and aspirin therapy but these did not increase following exercise. However, acute exercise resulted in a prothrombotic state evident in both groups, although this was more prolonged in patient with IC. The clinical significance of these findings in patients who are known to be at an increased risk of cardiac and other thrombotic event are unclear.

## Background

Regular, supervised, aerobic exercise has been shown to improve maximum walking distance and quality of life in patients with peripheral arterial disease and intermittent claudication (IC) [1]. However, current evidence suggests that exercise in patients with intermittent claudication may induce a systemic thrombo-inflammatory response [2,3]. Repeated bouts of muscle ischaemia induced by exercising to the onset of muscle pain, followed by reperfusion on rest, results in the generation of oxygen-derived free radicals (ODFR), subsequent neutrophil activation, release of arachadonic acid from membrane phospholipids, pro-inflammatory eicosanoids and cytokines [3-6].

ODFR, in particular the hydroxyl radical, cause lipid peroxidation, resulting in structural damage and activation of the vascular endothelium, increased vascular permeability and systemic capillary endothelial swelling [2,3,5]. A consequence of this altered capillary permeability is an increase in transcapillary leak of plasma proteins, resulting in increased renal albumin excretion [7]. Increases in urinary albumin excretion (expressed as the albumin creatinine ratio [ACR]) and protein creatinine excretion (PCR) have been demonstrated post- treadmill exercise in patients with intermittent claudication [7-10]. The increases in ACR have been shown to be attenuated by exercise therapy [9]. It is not clear if secondary prevention therapy, which includes aspirin and statin is able to attenuate the increase in glomerular permeability. The effect of exercise on tubular function is also unclear. N-acetyl-β-D-glucosaminidase (NAG) is a renal lysosomal enzyme found primarily in the proximal convoluted tubule. NAG is involved in the breakdown of glycoproteins [11]. Increased lysosomal turnover, such as that caused by proteinuria results in a small increase in urinary NAG. Larger increases represent renal, particularly tubular insult [12]. To the best of our knowledge, urinary NAG has not been assessed as a marker of renal dysfunction secondary to a possible exercise- induced inflammatory response.

In addition to exercise induced endothelial and leucocyte activation, there have been concerns that exercise may result in a pro-thrombotic state. Raised levels of various markers of coagulation such as D-dimer and thrombin-anti-thrombin complex (TAT) have been shown to occur in patients with intermittent claudication [13]. D-dimer is a breakdown product formed when plasmin acts on cross-linked fibrin. It may therefore be considered a marker of increased deposition of intra-vascular cross-linked fibrin (up regulation of coagulation cascade) or reflect up-regulation of the fibrinolytic pathway [14]. Raised levels of TAT complex in plasma are indicative of in-vivo thrombin generation [15]. To date, two studies have found an increase in thrombin generation following exercise, and only one of these showed an increase in fibrin breakdown in patients with IC who were not on statin therapy. Thus there is a possibility that exercise may lead to a pro-thrombotic state [16,17]. Statin therapy has been shown to reduce thrombin generation, and it is unclear what effect it may have on response to exercise in these patients [18]. In this study, we wished to determine if these observed changes in coagulation and inflammation would occur in patients maintained on statin therapy.

The aims of this observational study were to examine the effects of treadmill exercise on markers of inflammation, coagulation activation and renal function in patients with intermittent claudication on aspirin and statin therapy compared to healthy, age matched volunteers. The following parameters were measured: plasma interleukin-6 (IL-6), high sensitivity CRP (hsCRP), white blood cell count, thrombin-anti-thrombin complex (TAT) and fibrin D-Dimer (fDD), urinary protein creatinine ratio (PCR), ACR and NAG.

## Patients and methods

The study population consisted of 20 patients with intermittent claudication and 20 healthy volunteers (table 1). The local research ethics committee approved the study and written consent was obtained. Patients were required to have a diagnosis of chronic, stable intermittent claudication for at least six months and an ankle brachial pressure index (ABPI) of = 0.8. All patients were on secondary prevention therapy which included 75 mg of aspirin and statin therapy of at least six weeks duration. Statin therapy consisted of 40 mg of simvastatin or pravastatin or 10 mg of atorvastin, depending on an individual General practitioners prescribing practice. Patients were commenced on statin therapy irrespective of their initial baseline cholesterol level. Patients on clopidogrel, anti-inflamatory therapy, cilostazol or praxilene were excluded as were patients with diabetes. Patients who were judged unable to exercise due to other co-existing morbidities were excluded. The healthy age-matched volunteers were recruited from the surgical out-patient clinics. The presence of cardiovascular disease was excluded by medical questionnaire and physical examination including ABPIs. All volunteers had an ABPI greater than or equal to 1. No control subject was taking anti-platelet, anti-inflammatory, statin or steroidal therapy for at least 14 days prior to testing. The demographic details for the patient and volunteer group are shown in table 1.

**Table 1:** Patient and volunteer demographics

	**Volunteers (n = 20)**	**Claudicants (n = 20)**
Male:Female	11:9	16:4
Mean Age (SD)	63 (10)	68 (8)
Mean ABPI (SD)	1.14 (0.10)	0.62 (0.13)
Smokers		
Never	13	1
Reformed	5	12
Current	2	7
Aspirin Therapy	0	20

### Treadmill exercise

Patients with claudication walked on a treadmill (speed 3.5 km/h, incline 5 degrees) to their maximum walking distance. Exercise duration, time to onset and resolution of typical calf pain was recorded for all patients. Healthy controls walked for 3 minutes and 20 seconds which corresponded to the average patient exercise duration and represented a walking distance of 194 metres.

#### Blood Sampling and processing

Patients and volunteers attended for exercise after 9 am in the morning and were fasted for at least 6 hours having abstained from unaccustomed physical activity for the preceding 24 hours. All subjects rested for 30 minutes in a supine position prior to initial blood sampling. An 18G cannula was inserted into a large vein in the ante-cubital fossa. Patency was maintained by flushing the cannula with 5 ml boluses of 0.9% normal saline between sampling. Blood samples were collected using a "double syringe" technique and without the use of a tourniquet. The first 10 millilitres of blood collected was discarded. Blood was centrifuged (4 degrees centigrade at 2500 G) and the plasma separated, snap-frozen in liquid nitrogen and stored at -80 degrees centigrade for subsequent analysis. Further blood samples were collected immediately after the cessation of exercise and following a period of one hour of rest.

### Urine collection

On arrival, subjects emptied their bladder completely. After resting in a supine position for 30 minutes a mid-stream sample or urine was provided. Further samples were obtained in a similar manner 10 minutes after exercise and after 1 hour of recovery with complete voiding between each sample. Samples were frozen and stored at -20°C until analysis.

#### Analysis of samples

The following thrombo-inflammatory markers were measured at all timepoints: Plasma IL-6, HsCRP, TAT complex, Fibrin D-dimer, urinary protein, albumin and NAG. White cell count was assessed at baseline and immediately post-exercise.

### IL-6 assay

Plasma IL-6 was measured using a high sensitivity ELISA (Diaclone, Cedex, France). All samples were assayed in duplicate and all subjects' samples were analysed on the same run. The limit of detection was 1 pg/ml. The intra-assay coefficient of variation (CV) was 4.6%. The inter-assay CV was 6.7%.

### High-sensitivity CRP

HsCRP was measured using a commercially available rate nephelometric technique on a BN ProSpec analyser (Dade Behring Ltd, Milton Keynes, UK). The limit of detection was 0.175 mg/L CRP. Intra-assay CV was 3.5%. Inter-assay CV was 3.4%.

### White cell count (WBC)

White cell count was performed as part of an automatedfull blood count in the Haematology Laboratory, ARI, using the Bayer Advia 120 System (Bayer, Newbury, UK.)

### TAT complex

TAT was measured by ELISA (Enzygnost, Dade-Behring, Sysmex, Milton Keynes, UK). All samples were assayed in duplicate and all patient samples were analysed on the same run. Intra-assay CV was 3.6%. The inter-assay CV was 4.3%.

### Fibrin D-dimer

D-dimer was measured by immunoassay on the mini-Vidas instrument (Biomerieux UK Ltd, Basingstoke, UK). All samples were assayed in duplicate and all patient samples were analysed on the same run. Inter-assay CV was 4.7%.

### Urinary NAG

NAG was measured using a commercial colorimetric assay (PPR Diagnostics Ltd, London, UK) on a Roche Modular P system (Roche Diagnostics Ltd, East Sussex, UK). The limit of detection was 0.25 μmol/min/l NAG. Intra-assay CV was 3.9%. The inter-assay CV was 3.8%. NAG was expressed as a ratio with urinary creatinine so as to adjust for differences in urinary flow of the sample (i.e. μmol/h/mmol creatinine). The reference range is quoted as 0.12–0.47 μmol/min/mmol creatinine.

### Urinary protein

Urinary protein was measured using a turbidimetric technique on the Roche Modular P system (Roche Diagnostics Ltd, East Sussex, UK). The analyser was calibrated using commercially available human albumin/globulin standards of known concentration (Preciset U/CSF Protein, Roche Diagnostics GmbH, Germany). The limit of detection was 20 mg/l for urinary protein. The manufacturers quoted intra-assay C.V. was 1.0–5.2% and the inter-assay C.V. was 0.6–1.0%.

### Urinary albumin

Urinary albumin was measured using a nephelometric monoclonal antibody method on a BN Prospec analyser (Dade Behring Ltd, Milton Keynes, UK). The analyser was calibrated using N-Protein Standard and quality control samples were included in each run (Precinorm Albumin and Precipath Albumin, Roche Diagnostics Limited, Sussex, UK). The limit of detection was 1 mg/l for urinary albumin. The manufacturers quoted intra-assay C.V. was 1.2–1.6% and the inter-assay C.V. was 2.8–3.3%.

#### Urinary creatinine

Creatinine was measured using an optimised kinetic Jaffe reaction (32) on the Roche Modular P system (Roche Diagnostics Limited, Sussex, UK). The analyser was calibrated using C.f.a.s (Calibrator for automated systems) and standard controls were included in each run (Precinorm Albumin and Precipath Albumin, (Roche Diagnostics Limited, Sussex, UK). The limit of detection was 2 mmol/l and the manufacturers quoted intra-assay C.V. was 1.1–2.1%, inter-assay C.V. was 1.2–2.2 %. Both urinary protein and albumin concentrations were correlated for urine flow by factoring by creatinine concentration of the sample (i.e. mg/mmol creatinine). The technique of using single voided urine samples to estimate quantitative proteinuria is well validated and accurate [19].

### Statistical methods

All statistical analysis was performed using SPSS Version 11.5 for windows. All data were non-parametrically distributed. Initial analysis was via Friedmans test. Between time-point comparisons were performed using the Wilcoxon signed-rank test. Thus for each patient the post-exercise values were compared to their original baseline values and therefore each patient served as their own control. We acknowledge that there are a large number of possible confounding variables which could influence an individual patients response to exercise. However, it was felt that subgroup analysis of a patient cohort of 20 would be inappropriate. The difference in baseline measurements between groups was tested using the Mann-Whitney U-test. Chi-squared test was used to measure variation in demographic parameters between the groups. Expert statistical advice was sought from the Department of Statistics, University of Aberdeen.

## Results

The mean maximal walking time of the patients was 3 minutes 20 seconds (SD 130 seconds) which corresponded to a mean distance of 320 m. None of the healthy volunteers complained of lower limb pain during the exercise

### IL-6

In the healthy volunteers 78% of samples returned IL-6 values below the detectable range of the assay (<1.1 pg/ml). In patients with IC 42% of IL-6 levels were below the detectable range. For statistical analysis all values below the detectable range were given the value 0 pg/ml IL-6. Baseline IL-6 values were higher in patients with IC (p = 0.001). There was no change in plasma IL-6 concentration following exercise (table 2).

**Table 2:** Plasma markers of inflammation following exercise.

**Assay**	**Group**	**Baseline**	**Immediately post-exercise**	**1 hour post-exercise**	**P value**
**IL-6**	**HV**	0.0 (0.0–4.9)	0.0 (0.0–6.2)	0.0 (0.0–4.7)	>0.05
	**IC**	1.6 (0.0–15.7)	1.6 (0.0–12.3)	1.7 (0.0–15.3)	>0.05
**HsCRP**	**HV**	0.9 (0.2–16.2)	1.0 (0.2–13.5)	0.9 (0.2–12.6)	>0.05
	**IC**	2.9 (0.5–23.0)	3.0 (0.5–24.7)	2.9 (0.5–20.1)	>0.05

HV = healthy volunteers, IC = patients with intermittent claudication. Numbers represent medians with the range in brackets. P values represent Friedman test

### HsCRP

Baseline hsCRP values were higher in patients with IC compared to volunteers (p = 0.035). CRP levels were unaffected by exercise in the patients with IC or healthy volunteers (table 2).

### White Cell Count (WBC)

Baseline WBC was significantly higher in patients with IC compared to volunteers (p = 0.011). WBC increased significantly following exercise in both groups (p < 0.05). The median (range) increased from 7.6 (5.6–13.4) to 8.9 (7.1–17) immediately following exercise in patients with IC. Corresponding values for the volunteers prior to and immediately following exercise were 6.5 (4.4–11.0) and 7.8 (5.2–14) respectively.

### D-dimer

**Figure 1:**
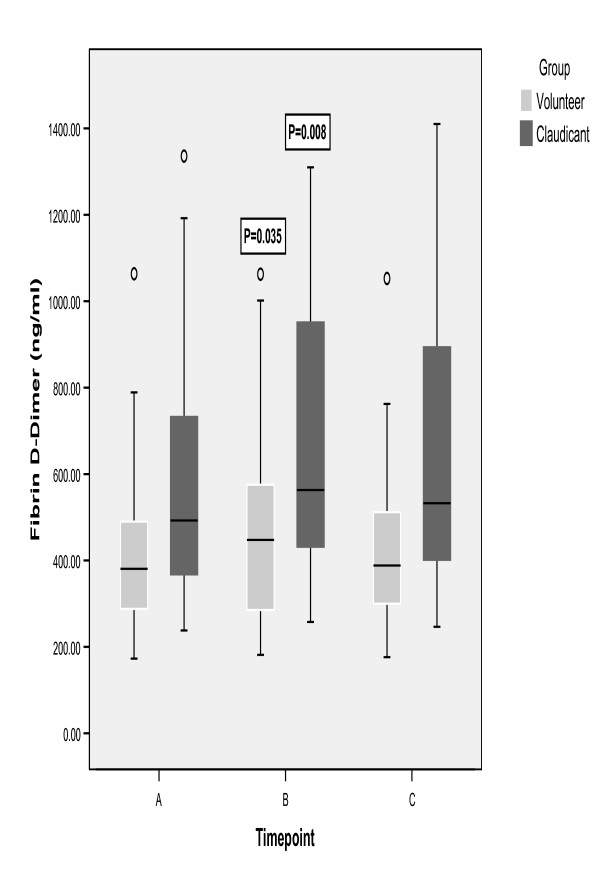
Plasma D-Dimer following treadmill exercise. A = baseline; B = immediately post-exercise; C = 1 hour post-exercise. Data presented as box-plots- line in centre of box represents the median value; the box represents the inter-quartile range and the whiskers represent the range. Markers out with the whiskers are outliers i.e. cases with values between 1.5 and 3 box lengths from the upper or lower edge of the box. P- values are Wilcoxon signed-rank test compared to baseline. Friedman test: P < 0.05

Fibrin D-dimer levels were significantly higher in patients with IC compared to healthy volunteers (median 520 ng/ml vs 381 ng/ml [p = 0.003]). Following exercise, D-dimer levels increased in both groups (Figure 1). Median percentage increase in volunteers was 7% (range -18% to 71% [p = 0.035]) and in patients with IC was 9% (range -7% to 88% [p = 0.008]). The magnitude of the changes in both groups was comparable. D-dimer levels remained elevated at 1 hour in the patients with IC.

### TAT-complex

**Figure 2:**
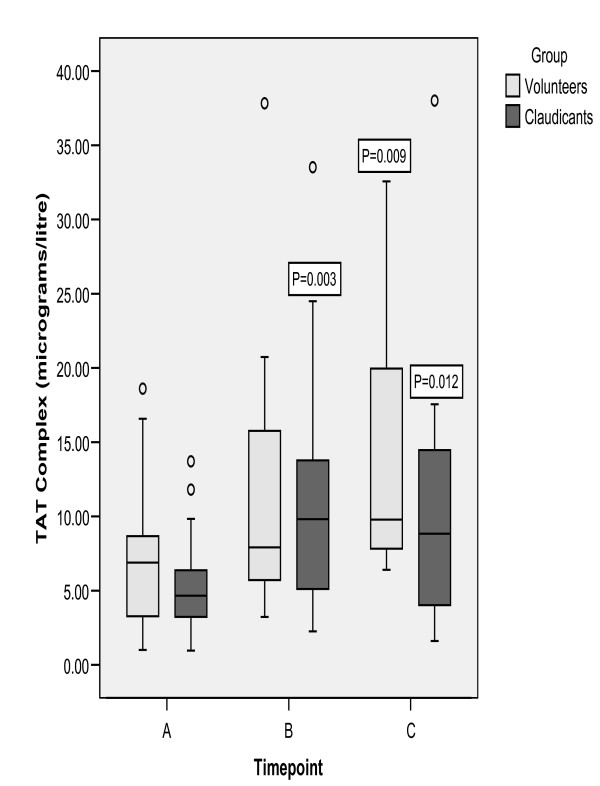
Plasma TAT complex following treadmill exercise. Healthy volunteers are represented by the grey boxes, and patients with intermittent claudication by the black boxes. A = baseline; B = immediately post-exercise; C = 1 hour post-exercise. Data presented as box-plots- line in centre of box represents the median value; the box represents the inter-quartile range and the whiskers represent the range. Markers out with the whiskers are outliers i.e. cases with values between 1.5 and 3 box lengths from the upper or lower edge of the box. P- values are Wilcoxon signed-rank test compared to baseline. Friedman test: P < 0.05

Baseline TAT levels did not differ between patients with IC and volunteers. TAT rose immediately post exercise in patients with IC and remained elevated at 1 hour (Figure 2). The median % change was 128% (range -43% to 400% [p = 0.003]). There were no immediate changes in TAT following treadmill exercise in volunteers, however, TAT levels were significantly elevated at 1 hour compared to baseline (p = 0.009) (Figure 2).

### Urinary PCR/ACR

Baseline PCR and ACR did not differ between the groups although there was a trend for higher urinary protein levels in patients with IC. After exercise PCR increased in both volunteers (p = 0.003) and patients with IC (p = 0.005). There was a significant increase in ACR following exercise in patients with IC only (p= 0.025) which returned to baseline values at 1 hour (table 3).

**Table 3:** Renal function following exercise.

**Assay**	**Group**	**Baseline**	**Immediately post-exercise**	**1 hour post-exercise**	**P value**
**PCR**	**HV**	7.0 (4.0–25.0)	9.0^† ^(5.0–34.0)	9 (5.0–31.0)	0.014
	**IC**	7.0 (2.0–52.0)	10.5^† ^(2.0–63.0)	7.5 (3.0–58.0)	0.005
**ACR**	**HV**	0.9 (0.9–13.2)	1.0 (0.9–19.6)	0.9 (0.9–20.2)	>0.05
	**IC**	1.0 (0.9–34.2)	1.75^‡ ^(0.9–34.7)	1.0 (0.9–31.3)	0.046
**NAG**	**HV**	0.7 (0.4–1.5)	0.8 (0.3–2.1)	0.8 (0.4–1.4)	>0.05
	**IC**	1.4 (0.2–7.8)	1.4 (0.2–6.8)	1.2 (0.2–5.0)	>0.05

HV = healthy volunteers, IC = patients with intermittent claudication. Numbers represent medians with the range in brackets. P values represent Friedman's test. ‡p < 0.05, †p < 0.001 (Wilcoxon signed rank test, comparisons made with baseline values).

### Urinary NAG

Patients with IC had higher baseline NAG levels compared to healthy volunteers (P = 0.011). There was no change in urinary NAG levels post-exercise (table 3).

## Discussion

In this study, elevated baseline levels of the inflammatory markers Hs-CRP, IL-6, and white cell counts were observed in patients with intermittent claudication despite statin and aspirin therapy. This chronic inflammatory state has previously been documented in patients with intermittent claudication who were not receiving both statin and aspirin therapy [20,23]. Recently concern has risen, that exercise in patients with intermittent claudication may result in yet a further increase in the level of these inflammatory markers [3].

In this study, the inclusion of age matched healthy controls allowed us to determine if any observed effects were due to exercise alone or due to a pro-inflammatory response induced by muscle ischaemia in patients with IC. Thus the duration of treadmill exercise in the volunteer group was limited to allow direct comparison with the patients with IC. The groups were not matched in relation to smoking and each patient and volunteer were used as there own control. We found no increase in Hs CRP or IL-6 following exercise in patients with IC on aspirin and statin therapy or in the healthy volunteers [24]. The effect of acute exercise on high sensitivity CRP has not been reported before, but exercise training has been shown to reduce levels of CRP [2,25]. Only one previous study has examined the effect of exercise on IL-6 levels, a precursor of CRP, and shown this to be increased pre and post-exercise in 20 patients with IC compared to 20 healthy controls [6]. These patients were not on statin therapy which has been shown to reduce IL-6 and CRP production in the endothelial and liver cells [26,27].

Despite the lack of inflammatory response, exercise did appear to result in an imbalance between the thrombotic and fibrinolytic pathways in both patients with IC and healthy controls. We have previously shown TAT levels to be increased in patients with critical limb ischaemia, but not in patients with IC compared to controls [13]. Similarly, in this study, baseline TAT levels did not differ between patients with IC and healthy volunteers despite the fact that there was a much higher preponderance of smokers in the patient group. D-dimer baseline levels were elevated as would be expected in the patient group [13,28,29]. Following treadmill exercise TAT levels increased in the patients with IC and in volunteers. However, the increase in TAT levels was immediate and more prolonged in the patients with IC. In healthy volunteers changes in TAT levels have been shown to vary depending on the nature of exercise and fitness of the individual, but it is surprising to observe a significant rise in TAT at 1 hour following limited exercise [30,31]. A rise in D-dimer levels was also observed in both groups suggesting concomitant up-regulation of the fibrinolytic pathway, but this was only evident immediately after exercise. Thus the pro-thrombotic effect of exercise, as assessed by TAT levels was more prolonged. Mustonen et al [17], showed exercise induced increases in both TAT and D-dimer, whereas a smaller study by Burns et al showed an increase in TAT but not D-dimer post-exercise in patients with IC [16]. The findings of the later study, like ours suggest an imbalance in the fibrinolytic system and a pro-thombotic state. This finding which is more pronounced in the patient group has occurred despite the use of statins which have been shown to reduce thrombin generation [18]. While it would have been desirable to have assessed the effect of exercise on these pro-thrombotic markers in patients with IC who are not on aspirin and statin therapy this would be difficult to achieve and indeed unethical. It would also not be relevant to current accepted practice of the management of patients with IC. The effect, of exercise training on TAT levels are unknown, but a recent study has shown that exercise therapy results in a reduction in the levels of PAI-1 an inhibitor of fibrinolysis in patients with intermittent claudication [33]. However, the recent suggestion that successful lower limb angioplasty may reverse any thrombo-inflammatory effects of exercise seen in patients with IC may be inappropriate given the changes observed in the volunteer group [10].

Importantly, this study is the first to assess the effect of exercise on urinary NAG which is a sensitive index of subclinical proximal tubular damage [34]. NAG was elevated in patients with intermittent claudication compared to volunteers at baseline suggesting on-going renal insult in patients with intermittent claudication. Increases in urinary NAG excretion have been demonstrated in patients undergoing aortic surgery [35] and in patients with critical limb ischaemia [36] but has not previously been documented in patients with IC. Reassuringly, exercise in patients with IC did not result in a rise in urinary NAG. The baseline PCR and the ACR, which are markers of glomerular permeability, were similar in patients with IC and volunteers. The PCR increased in both groups immediately post-exercise but returned to baseline levels at 1 hour. The ACR, increased only in the patient with IC immediately following treadmill exercise and returned to baseline levels at 1 hour. The clinical significance of this increase in ACR is unclear but it has been shown to be attenuated following exercise training [9]. While the rise in PCR and ACR post exercise in patients with IC has previously been documented [7-10,36] this study has demonstrated how transient this phenomenon is in patients with IC and emphasises that an increase in glomerular permeability also occurs in healthy volunteers.

We conclude that in patients with intermittent claudication on secondary prevention therapy including aspirin and a statin, there is evidence of an on going chronic inflammatory response. However, in these patients exercising to maximum walking distance did not result in a rise in the inflammatory markers HsCRP or IL-6. Exercise resulted in a transitory increase in glomerular permeability but no change in renal tubular function. Exercise did result in increased TAT levels suggesting in-vivo thrombin generation in both patients with IC and healthy volunteers, but the effect in patients was immediate and more prolonged. The increase in D-dimer levels was more transitory suggesting that an imbalance in the thrombotic and fibrinolytic pathway may exist despite statin and antiplatelet therapy. Currently, the clinical significance of this mismatch in the thrombotic and fibrinolytic system is unclear and further studies are required. However, its occurrence in patients with IC who have an increased risk of adverse thrombotic events may be a cause for concern.
